# Salivary Micro-RNA and Oral Squamous Cell Carcinoma: A Systematic Review

**DOI:** 10.3390/jpm11020101

**Published:** 2021-02-04

**Authors:** Maria Menini, Emanuele De Giovanni, Francesco Bagnasco, Francesca Delucchi, Francesco Pera, Domenico Baldi, Paolo Pesce

**Affiliations:** 1 Division of Prosthodontics and Implant Prosthodontics, Department of Surgical Sciences (DISC), University of Genova, 16126 Genova, Italy; lele9-90@hotmail.it (E.D.G.); fcbagna5@hotmail.it (F.B.); dafne.1995@libero.it (F.D.); domenico.baldi@unige.it (D.B.); paolo.pesce@unige.it (P.P.); 2 Department of Surgical Sciences, CIR-Dental School, University of Turin, 10126 Turin, Italy; francesco.pera@unito.it

**Keywords:** oral squamous cell carcinoma, miRNAs, diagnosis, prognosis, saliva, biomarker

## Abstract

Oral squamous cell carcinoma (OSCC) is a widespread malignancy with high mortality. In particular, a delay in its diagnosis dramatically decreases the survival rate. The aim of this systematic review was to investigate and summarize clinical results in the literature, regarding the potential use of salivary microRNAs (miRNAs) as diagnostic and prognostic biomarkers for OSCC patients. Twelve papers were selected, including both case–control and cohort studies, and all of them detected significantly dysregulated miRNAs in OSCC patients compared to healthy controls. Based on our results, salivary miRNAs might provide a non-invasive and cost-effective method in the diagnosis of OSCC, and also to monitor more easily its evolution and therapeutic response and therefore aid in the establishment of specific therapeutic strategies.

## 1. Introduction

Oral cavity cancer is the most frequent malignancy of the head and neck. It represents the 16th most common malignancy and the 15th leading cause of death worldwide, with an incidence of oral cancer (age adjusted) in the world of four cases per 100,000 people, with a wide variation across the globe which depends on gender, age groups, countries, races and ethnic groups and socio-economic conditions [1].

Roughly 90% of oral cancers histologically originate from squamous cells and are classified as oral squamous cell carcinoma (OSCC). OSCC develops in the oral cavity and oropharynx and can occur due to many etiological factors; smoking and alcohol remain the most common risk factors especially in the western world. Other risk factors include diet, immunodeficiency and high-risk HPV 16/18 [2]. There are also several genetic alterations involved in oral carcinogenesis. Among these, alterations in oncosuppressors (APC, p53), proto-oncogenes (Myc), oncogenes (Ras), and genes that control normal cellular processes (EIF3E, GSTM1) play a fundamental role in cancer development [3].

Despite advances in therapies, the overall 5-year survival rate has remained unchanged during the past decades. While at early stages the survival rate is approximately 89%, at late stages it decreases to 39%. Unfortunately, oral cancer patients are still frequently diagnosed in advanced stages despite educational interventions for prevention and early diagnosis, that is the most important prognostic factor for predicting survival [4].

To date, the gold standard for OSCC diagnosis is represented by a clinical oral examination integrated by a histological investigation on tissue biopsies of suspicious lesions [5]. However, cancer research is currently focusing on finding less invasive and cost-effective methods to provide a more comprehensive view of the cancer profile, also to more easily monitor its evolution and therapeutic response and therefore aid in the establishment of specific therapeutic strategies [6]. MicroRNAs (miRNAs) might provide a useful tool in this regard.

MiRNAs are small noncoding RNAs (ncRNAs) of approximately 22 nucleotides responsible for specific regulation of gene expression in a post-transcriptional manner. They are the main regulator of gene transcription and bear relevance in predicting clinical outcomes. Indeed, only less than 5% of expressed genes producing messenger RNA is really translated into proteins while miRNAs are fully functionally active in cell cytoplasm; they are responsible for various cellular and metabolic pathways, including cell proliferation, differentiation, and survival [7,8]. Deregulation of miRNAs has been reported in a number of diseases. In Implant Dentistry miRNAs have been found to be predictors of dental implants clinical outcomes and may be used as biomarkers for diagnostic and prognostic purposes [9,10].

In recent years, researchers have found that miRNA expression is dysregulated in human malignant tumor cells [11]. Due to their stability in human peripheral blood and body fluids and disease specific expression, an increasing number of studies indicate that miRNAs may represent an ideal set of biomarkers applied in early diagnosis and prognosis of cancers [12]. Related to OSCC, a growing number of studies has demonstrated that certain miRNAs are differentially expressed in oral cancer and analyses have indicated that the differentially expressed miRNAs may help distinguishing patients with oral cancer from healthy subjects [13]. In addition, dysregulation due to distinct polymorphisms in mature miRNAs, particularly miR-196a2 rs11614913 C>T, miR-146a rs2910164 G>C, miR-149 rs2292832 C>T, and miR-499 rs3746444 A>G, are associated with the risk of OSCC [14,15]. Several recent systematic reviews focused on the role of miRNAs in oral cancer. These papers show that miRNAs may assist in the prognosis of head and neck cancer and they can also predict resistance to chemotherapeutic agents, recurrence and metastasis [16,17,18]. Most of the reviews focused on oral cancer in general [19,20] or the evaluation of circulating miRNAs from serum or plasma [17,18]. Repetitive blood sampling can often be time consuming and physically intrusive, adding excessive stress and pain to patients, thereby leading to poor patient compliance. In the last years, salivary diagnostics has attracted significant attention among clinicians and scientists since the method of sample collection for disease is cost-effective, accurate and noninvasive. Moreover, oral cancer cells are immersed in the salivary milieu. Unlike other kinds of body fluid, oral tissues are continually immersed in saliva. Therefore, saliva may provide direct information regarding the disease status of oral mucosa. Saliva has been used as a diagnostic medium for OSCC, and saliva analytes such as proteins and DNA have been used to detect OSCC [21]. Thousands of miRNAs are present in saliva, and a panel of salivary miRNAs can be used for oral cancer detection [22]. Salivary miRNAs appear to enter the oral cavity through various sources, including the three major saliva glands, gingival crevice fluid, and desquamated oral epithelial cells. The majority of salivary miRNAs appear to be present as partially degraded forms. These partially degraded miRNAs maintain their stability in saliva through their association with unidentified macromolecules [23].

The aim of the present systematic review was to investigate and summarize results in the literature, regarding the potential use of salivary miRNAs as diagnostic and prognostic biomarkers for OSCC patients. In particular, the salivary miRNAs differently expressed in the saliva of patients with OSCC compared to healthy subjects were investigated.

## 2. Materials and Methods

The present systematic review was conducted according to guidelines reported in the indications of the Preferred Reporting Items for Systematic Review and Meta-Analysis (PRISMA) [24].

The focused question was: “What are the miRNAs differently expressed in the saliva of patients with oral squamous cell carcinoma (OSCC) compared to patients without OSCC?”

### 2.1. Search Strategy

The following Internet sources were used to search for papers that satisfied the study purpose: the National Library of Medicine (MEDLINE—PubMed), Scopus and Cochrane Library. In addition, a partial research of the gray literature was carried out through Google Scholar. The last search was done on 8 December 2020. We used the following search terms to search all databases: microRNA, microRNAs, miRNA, miRNAs, miR, mi-RNA combined with “oral squamous cell carcinoma” or OSCC.

All the clinical studies investigating miRNAs in patients with OSCC were included if they met the following inclusion criteria:

- Patients diagnosed with OSCC;
- Minimum of 10 patients included;
- Study subjects included cancer patients and healthy controls;
- Possibility to extrapolate data for patients with OSCC (data regarding oral cancer in general or head and neck cancer were excluded).

Eligible articles included: cross-sectional, case–control and cohort studies. Publications that did not report salivary miRNAs and their role as diagnostic/prognostic biomarkers in OSCC and did not meet the above inclusion criteria were excluded. Papers that were not dealing with original clinical cases (e.g., reviews, conference abstracts, personal opinions, editorials, etc.) and multiple publications from the same pool of patients (redundant publications) were also excluded. No restrictions in terms of year or language of publication were applied. No publication status restrictions were imposed. In addition, full-text articles of narrative and systematic reviews published between 2018 and 2020 and dealing with miRNAs and oral cancer were obtained. A hand search was performed by screening these reviews and the reference list of all included publications.

### 2.2. Screening and Selection

Titles and abstracts of the searches were screened by two independent reviewers (EDG and MM) for possible inclusion. Disagreements between reviewers were resolved by discussion between the two review authors; if no agreement could be reached, a third author decided (PP). The full texts of all studies of possible relevance were then obtained for independent assessment by the reviewers. If title and abstract did not provide sufficient information to determine eligibility of the study, the full report was obtained as well.

### 2.3. Data Extraction

Data from the studies included in the final selection were extracted by one of the authors using Microsoft Excel spreadsheet software (Excel 16.4, Microsoft CO, Redmond, WA, USA) (EDG). The accuracy of data was verified independently by another coauthor (FD). The following data were extracted: author(s), publication year, title, study design, nation where the study was conducted, sample size of both cases and controls (individuals with OSCC and healthy subjects respectively), diagnostic stage of disease, follow-up period, miRNAs detection method, name of the miRNA(s) identified as dysregulated, type of dysregulation recorded and main outcomes.

### 2.4. Quality Assessment

The risk of bias of included studies was assessed using the Newcastle Ottawa scale (NOS). Two reviewers (EDG, FB) independently evaluated the quality of studies based on the following parameters: Selection, Comparability, and Outcome/Exposure. A maximum of 4 stars in selection domain, 2 stars in comparability domain and 4 stars in outcome/exposure domain were given. The included studies were qualified as “Good”, “Fair” and “Poor” quality based on the total NOS score they achieved. Studies with a NOS score ≥ 7 were considered good-quality studies.

## 3. Results

### 3.1. Bibliographic Search and Study Selection

**Figure 1:**
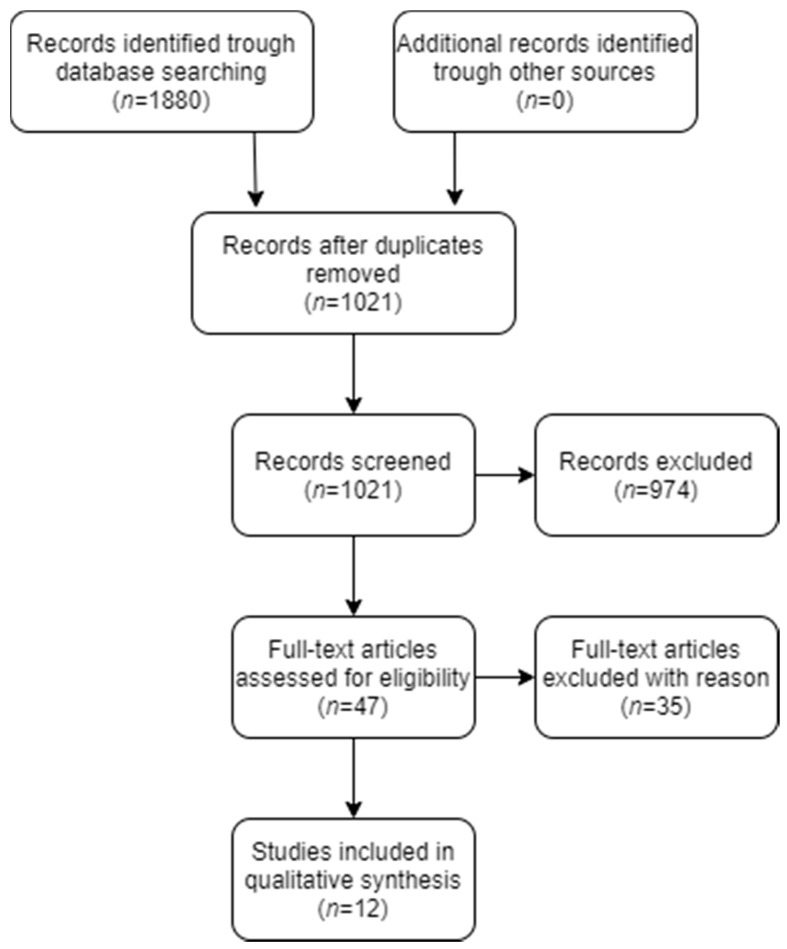
Preferred reporting of systematic reviews and meta-analyses (PRISMA) flow diagram related to bibliographic searching and study selection.

The initial database search yielded a total of 1880 entries; of which 977 were found in PubMed^®^/MEDLINE, 898 in Scopus, and five in Cochrane library. In addition, a partial research of the gray literature was carried out through Google Scholar. A flow chart that depicts the screening process is displayed in Figure 1. After excluding all duplicates, the total number of entries was reduced to 1021. A total of 974 articles were excluded after review of title and abstract. Hence, full-text examination was conducted for 47 articles. A total of 35 additional articles were excluded after full-text review and application of the eligibility criteria. The final selection consisted of 12 articles. Detailed data for the 12 included studies are listed in Table 1. 

### 3.2. Description of Included Studies

Detailed data for the 12 included studies are listed in Table 1. All studies included in the present review are studies performed on humans.

Two studies were conducted in USA [25,28]; two in Australia [32,35]; one in Denmark [26]; one in Taiwan [27]; one in Turkey [30]; one in Iran [33]; one in Korea [31], one in Italy [34] one in Egypt [29], and one in China [36].

All the studies utilized real time quantitative polymerase chain reaction (RTq-PCR) to quantify salivary miRNAs. Three studies (Duz et al. 2016; Seung-Ki Min et al. 2017; He L et al. 2020) also utilized microarray-based miRNA analysis.

All the papers included identified a set of significantly dysregulated miRNAs in OSCC patients compared to healthy controls. Different types of miRNAs were found to be upregulated and other downregulated in OSCC patients. In particular, mir21 was identified as upregulated in OSCC patients compared to healthy controls in three studies (Zahran et al. 2015; Yap T et al. 2018; Mehdipour et al. 2018); and mir31 was identified as upregulated in OSCC patients compared to healthy controls in other three papers (Liu et al. 2012; Yap T et al. 2018; Mehdipour et al. 2018).

Two studies (Park et al. 2009; Mehdipour et al. 2018) identified mir200a and mir125a as downregulated in OSCC patients compared to healthy controls.

The other studies did not report superimposable outcomes.

### 3.3. Excluded Studies

Out of 47 papers for which the full-text was analyzed, 35 articles [37,38,39,40,41,42,43,44,45,46,47,48,49,50,51,52,53,54,55,56,57,58,59,60,61,62,63,64,65,66,67,68,69,70,71]. were excluded from the systematic review. (Table A1). The main reasons for exclusion were the following: study type; miRNAs detected in samples different from saliva and data regarding not OSCC but oral cancer in general or premalignant conditions.

### 3.4. Quality Assessment of Included Studies

The risk of bias of included studies was assessed using the Newcastle Ottawa scale (NOS). Outcomes are reported in Table 2.

## 4. Discussion

The 5-year survival rates in OSCC depends on the stage at diagnosis. Patients have better survival and favorable outcomes if detected early, as compared to those diagnosed in advanced stages. There are several screening and awareness programs implemented, but they have not been able to lower the incidence of OSCC.

Traditional diagnostics for malignant tumors such as tissue biopsy and mucosal scraping examination can often be time-consuming and physically intrusive, adding excessive stress and pain to patients, thereby leading to poor patient compliance. In this context, the addition of a non-invasive diagnostic test based on a liquid biopsy would be beneficial in the early diagnosis and prognosis of such diseases as an alternative to solid biopsies. Liquid biopsy is a novel approach that relies on the study of circulating cells, circulating DNA, micro-RNA, microvesicles and exosomes in body fluids supporting the concept of personalized medicine [72,73]. In recent decades, saliva has been widely investigated as a promising source of OSCC biomarkers for liquid biopsy [74].

There are many advantages to employing saliva as a substrate for diagnostic analysis. Its sampling is inexpensive and non-invasive. Aberrant expression profiles of salivary miRNAs have been detected in different types of cancer, showing their power as a discriminatory clinical method [75]. A recent systematic review and meta-analysis demonstrated a high diagnostic accuracy of salivary and blood miRNAs to differentiate OSCC patients from healthy individuals, with sensitivity, specificity, and AUC (area under the SROC curve) values of 0.78, 0.82, and 0.91, respectively [43]. However, considerable heterogeneity was detected among the included studies and the authors suggested the need for further research on the topic.

A total of 12 papers were included in the present systematic review. Four of them were considered of good quality (NOS score equal to 7), and the remaining eight studies were considered at high risk of bias (NOS score 6 or 5), demonstrating the need for further studies with a more rigorous design on the topic.

The studies included applied heterogeneous methodologies to investigate the role of miRNAs in OSCC patients. In particular, the time of sampling (i.e., before and/or after surgery/radiotherapy) was not reported in all the studies. The research protocols included stimulated or unstimulated saliva for analysis of either whole saliva or salivary supernatant. In addition, salivary samples were taken at different clinical stages of OSCC and the studies differed regarding patients’ demographic characteristics (i.e., smoke, alcohol consumption, positivity to HPV, patients’ age and sex, etc.) and possible comorbidities possibly affecting the outcomes. Some of the studies included did not account for such patients’ characteristics nor for the site of OSCC (buccal mucosa, gingiva, tongue, etc.). For this reason, the realization of a metanalysis was contraindicated and it is difficult to draw comprehensive conclusions on the topic. These heterogeneities might also explain why the type of miRNAs identified and the dysregulations detected in the various studies were mostly not superimposable.

Differently from the other investigations included, the study by He et al. analyzed miRNAs in salivary exosomes. The lipid bilayer of exosomes can protect miRNAs from degradation by RNase in body fluids. However, the use of salivary exosomal miRNAs as biomarkers for human disease remains controversial. A critical limitation of using salivary exosomes for cancer screening is that differences in isolation techniques may alter the composition of purified subpopulations and the purity of the exosome pellets. Gai et al. analyzed miRNAs in salivary extracellular vesicles (EVs, including exosomes, microvesicles or ectosomes, and apoptotic bodies). A previous study [76]. showed that most of the salivary RNA was associated with EVs. However, miRNAs can be differentially represented in whole saliva and salivary EVs, as it has been previously described for total plasma or plasma-derived EVs [77,78].

Despite the limitations listed above, our data overall demonstrate that there is strong evidence for a role of miRNAs in OSCC behavior. In fact, all the 12 papers included detected significantly dysregulated miRNAs in OSCC patients compared to healthy controls. In particular, some miRNAs were identified as differently expressed in OSCC patients in different papers: mir21, mir31, mir200a and mir125a. This panel of four miRNAs appears to have a significant predictive value in OSCC. Further longitudinal studies are needed in order to confirm which specific salivary miRNAs are the most effective biomarkers for the diagnosis and management of OSCC patients.

In conclusion, the present systematic review suggests that salivary miRNAs might provide a non-invasive and cost-effective method in the diagnosis of OSCC, and also to monitor more easily its evolution and therapeutic response and therefore aid in the establishment of specific therapeutic strategies.

## Figures and Tables

**Table 1:** Main characteristics of the included studies.

Authors (Year)	Cases (OSCC Patients)	Controls (Healthy Subjects)	Tumor Histological Stage or Grade	miRNAs Detection Method	Salivary miRNAs—Disregulation in OSCC Patients	Application	Follow-Up	Main Outcomes
Park et al. 2009 [25].	50 OSCC	50	10 patients were at tumor stage I, 14 were at stage II, 16 were at stage III, 10 were at stage IV	RT-PCR preampqPCR Saliva miRNA stability assay	miR142-3p miR200a miR125a miR-93 Downregulation	Diagnosis	ND	miRNAs are present in both whole saliva and supernatant saliva. miR-125a and miR-200a, are downregulated in the saliva of OSCC patients compared to healthy controls
Wiklund et al. 2011 [26].	15 OSCC	7	ND	TaqManH low density array qRT-PCR	miR-34b miR-137 miR-155 miR-200c-141 miR-203 mir-205 miR-375 mir-410 Aberrant expression and DNA hypermethylation	Diagnosis	ND	Compared to healthy subjects, OSCC patients had deregulated miRNAs with associated DNA methylation patterns. Particularly, repression of miR-375 and methylation on miR-137, miR-200c-141, and miR-200 s/miR-205 loci were found in OSCC patients vs. healthy patients, being promising candidates to develop OSCC-specific miRNA signatures.
Liu et al. 2012 [27].	45 OSCC	24	21 stage I-II 24 stage III-IV	qRT-PCR	miR-31 Upregulation	Diagnosis and follow-up	4–6 weeks after surgery	Salivary miR-31 was significantly increased in patients with OSCC at all clinical stages, including very early stages. In addition, it was shown to be more abundant in saliva than in plasma, and after tumor surgical removal its expression was reduced.
Momen-Heravi et al. 2014 [28].	9 OSCC (before treatment), 8 OSCC-r (in remission)	9	ND	RT-qPCR	miR136 miR147 miR1250 miR148a miR632 miR646 miR668 miR877 miR503 miR220a miR323-5p underexpressed miR-24 miR27b overexpressed	Diagnosis	ND	miRNA profiles derived from OSCC, OSCC-r, and healthy controls were distinctively different. In particular, overexpression of miRNA-27b was found in OSCC saliva samples and not in the saliva of the other two groups
Zarhan et al. 2015 [29].	100 Oral cancer (20 OSCC)	20	Grade III (high grade) LN involvement: 7 Grade II, LN involvement (2 LN): 1 Grade II, no LN involvement: 7 No record available: 3	qRT-PCR	miR-21 upregulation miR184 upregulation miR145 downregulation	Diagnosis	ND	Salivary miRNA-21, miRNA-145, and miRNA-184 were differentially expressed in OSCC and healthy saliva samples, with miRNA-184 having the best diagnostic value
Duz et al. 2016 [30].	25 OSCC	25	2 grade 1 16 grade 2 4 grade 3 3 ND	qRT-PCR Microarray-based miRNA	miR139-5p downregulation	Diagnosis and follow-up	4–6 weeks after surgery	Salivary miR-139-5p was significantly reduced in TSCC patients compared to controls, and its level turned back to normal after surgery.
Seung-Ki Min et al. 2017 [31].	18 OSCC	ND	ND	RT-qPCR miRNA Microarray	miR-146a-5p upregulated	Diagnosis	ND	miR-146a-5p expression was highly upregulated in OSCC patients.
Yap T et al. 2018 [32].	30 OSCC	30	14 stage 1 3 stage 2 3 stage 3 10 stage 4	RT-qPCR	miR-24 miR-21 miR-31 upregulation miR-99a let-7c miR-100 downregulation	Diagnosis	ND	Upregulation of miR-31 and miR-21 and downregulation of miR-99a, let-7c, miR-125b, and miR-100 were found in OSCC and controls in both FFPE and fresh-frozen samples. These miRNAs were studied in oral swirls to develop a dysregulation score with the classification tree identifying 100% (15/15) of OSCC and 67% (10/15) of controls.
Mehdipour et al. 2018 [33].	30 OLP 15 OSCC	15	ND	qRT-PCR	miR-21 upregulation miR-125a downregulation miR-31 upregulation miR-200a downregulation	Diagnosis	ND	miR-21 levels were significantly increased in saliva samples derived from patients with OLP, dysplastic OLP and OSCC, compared to those from healthy controls. Conversely, significant decreases in miR-125a levels were found in the OLP, dysplastic OLP and OSCC samples, compared to those from healthy controls. Significant increases in miR-31 levels were found in samples derived from dysplastic OLP and OSCC patients, but not in nondysplastic OLP, compared to healthy controls. miR-200a levels were significantly decreased only in samples derived from OSCC patients
Gai C et al. 2018 [34].	21 OSCC	11	T1 (n = 7), T2 (n = 8), T3 (n = 3), T4 (n = 3)	qRT-PCR array qRT-PCR	miR-412- 3p, miR-489-3p, miR-512-3p, miR-597-5p, and miR-603 upregulated miR-193b-3p, miR-30e3p, miR-376c-3p, miR-484, miR-720, and miR-93-3pdownregulated	Diagnosis	ND	miR-302b-3p and miR-517b-3p were expressed only in EVs from OSCC patients and miR-512-3p and miR-412-3p were up-regulated in salivary EVs from OSCC patients compared to controls with the ROC curve showing a good discrimination power for OSCC diagnosis
Yap T et al. 2019 [35].	53 OSCC	54	24 T1 10 T2 3 T3 15 T4a 1 not specified	RT-qPCR	miR-24-3p, miR-21-5p, let-7c-5p, miR-99a-5p, miR-100-5p	Diagnosis	ND	MicroRNAs can be predictably isolated from oral swirls. A high-risk dysregulation signature was found to be accurate in indicating the presence of OSCC with 86.8% sensitivity and 81.5% specificity
He L et al. 2020 [36].	49 OSCC	14	ND	Microarray analysis qRT-PCR	miR-7975, miR-1246 and miR-24-3p upregulated	Diagnosis	ND	The authors identified a significant increase of miR-24-3p in the salivary exosomes from 45 preoperative OSCC patients compared to healthy individuals

OSCC—Oral squamous cell carcinoma; ND—Undeclared; OLP—Oral lichen planus; qRT-PCR—Quantitative reverse transcription polymerase chain reaction; RT-qPCR—Real-time quantitative polymerase chain reaction; qPCR—Quantitative polymerase chain reaction; RT-preamp-qPCR—reverse transcriptase preamplification-quantitative; FFPE—formalin-fixed paraffin-embedded; EV—extracellular vesicle.

**Table 2:** Risk of bias of included studies.

Study	Selection	Comparability	Outcome/Exposure	NOS Score
Park et al. 2009	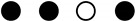	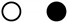	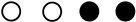	6
Wiklund et al. 2011	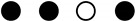	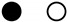	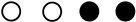	6
Liu et al. 2012	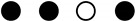	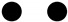	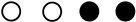	7
Momen-Heravi et al. 2014	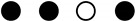	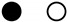	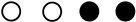	6
Zarhan et al. 2015	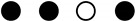	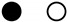	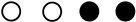	6
Duz et al. 2016	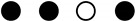	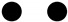	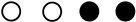	7
Yap T et al. 2018	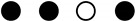	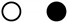	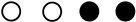	6
Mehdipour et al. 2018	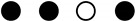	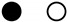	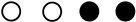	6
Gai C et al. 2018	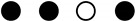	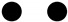	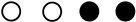	7
Yap T et al. 2019	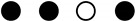	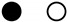	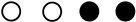	6
He L et al. 2020	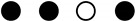	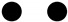	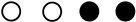	7
Seung-Ki Min et al. 2017	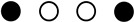	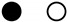	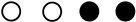	5

NOS-Newcastle Ottawa Scale.

## Appendix A

**Table A1:** Table reporting the 35 excluded studies and reasons for exclusion.

Article Excluded	Reason for Exclusion
Dumache R et al. 2017	Review article
Cristaldi M et al. 2019	Review article
Gaba et al. 2018	Review article
Salazar-Ruales et al. 2018	Oral cancer in general
Lay YH et al. 2018	In vitro study
Arantes et al. 2018	Review article
Rapado-Gonzalez et al. 2019	Review article
Wan Y et al. 2017	Oral cancer in general
Greither T et al. 2017	No control group
Fadhil et al. 2020	Oral cancer in general
Yeh LY et al. 2015	Oral cancer in general
Pentenero et al. 2019	Review article
Coon J et al. 2020	In vitro study
Brinkmann O et al. 2011	Review article
Langevin S et al. 2017	In vitro study
Shaidi M et al. 2017	Oral lichen planus
Dharmawardana N et al. 2019	Review article
Dumache R et al. 2015	Review article
Peacock B et al. 2018	Tissue sample
Patil S et al. 2019	Review article
Li et al. 2018	Tissue sample
Sun C et al. 2018	Tissue sample
Salazar C et al. 2014	Oral cancer in general
Gallo A et al. 2013	Descriptive article
Chen M et al. 2020	In vitro study
Yang et al. 2013	Oral premalignant lesion
Al Makey et al. 2015	Oral cancer in general
Hung et al. 2016	Oral premalignant lesion
Maheswari et al. 2020	Oral premalignant lesion
Petronacci et al. 2019	Tissue sample
Wang Y et al. 2018	Tissue sample
Pedersen et al. 2018	Tissue sample and plasma
Gissi et al. 2018	Tissue sample
Yang et al. 2017	Tissue sample
Moratin et al. 2016	Tissue sample
